# Insulin-Like Growth Factor Binding Protein 2 Is Associated With Biomarkers of Alzheimer’s Disease Pathology and Shows Differential Expression in Transgenic Mice

**DOI:** 10.3389/fnins.2018.00476

**Published:** 2018-07-16

**Authors:** Luke W. Bonham, Ethan G. Geier, Natasha Z. R. Steele, Dominic Holland, Bruce L. Miller, Anders M. Dale, Rahul S. Desikan, Jennifer S. Yokoyama, Alzheimer’s Disease Neuroimaging Initiative

**Affiliations:** ^1^Department of Neurology, Memory and Aging Center, University of California, San Francisco, San Francisco, CA, United States; ^2^Department of Radiology and Biomedical Imaging, University of California, San Francisco, San Francisco, CA, United States; ^3^Department of Neurosciences, University of California, San Diego, San Diego, CA, United States; ^4^Department of Psychiatry, University of California, San Diego, San Diego, CA, United States; ^5^Department of Radiology, University of California, San Diego, San Diego, CA, United States

**Keywords:** IGFBP-2, Alzheimer’s disease, CSF, neuroimaging, tau

## Abstract

There is increasing evidence that metabolic dysfunction plays an important role in Alzheimer’s disease (AD). Brain insulin resistance and subsequent impairment of insulin and insulin-like growth factor (IGF) signaling are associated with the neurodegenerative and clinical features of AD. Nevertheless, how the brain insulin/IGF signaling system is altered in AD and the effects of these changes on AD pathobiology are not well understood. IGF binding protein 2 (IGFBP-2) is an abundant cerebral IGF signaling protein and there is early evidence suggesting it associates with AD biomarkers. We evaluated the relationship between protein levels of IGFBP-2 with cerebrospinal fluid (CSF) biomarkers and neuroimaging markers of AD progression in 300 individuals from across the AD spectrum. CSF IGFBP-2 levels were correlated with CSF tau levels and brain atrophy in non-hippocampal regions. To further explore the role of *IGFBP2* in tau pathobiology, we evaluated the expression of *IGFBP2* in different human and mouse brain cell types and brain tissue from two transgenic mouse models: the P301L-tau model of tauopathy and TASTPM model of AD. We observed significant differential expression of *IGFBP2* in both transgenic mouse models relative to wild-type mice in cortex but not in hippocampus. In both humans and mice, *IGFBP2* is most highly expressed in astrocytes. Taken together, our findings suggest that IGFBP-2 may be linked to tau pathology and provides further evidence for a relationship between metabolic dysregulation and neurodegeneration. Our results also raise the possibility that this relationship may extend beyond neurons.

## Introduction

Alzheimer’s disease (AD) is the most common cause of dementia, affecting more than 37 million people worldwide (Matthews, 2010). The pathological hallmarks of AD are insoluble extracellular amyloid beta plaques and intracellular neurofibrillary tangles (NFTs) containing aggregates of hyperphosphorylated tau (p-tau) protein (Perl, 2010; Querfurth and LaFerla, 2010). This pathology is associated with neuronal cell loss and synaptic injury that leads to the characteristic memory loss, cognitive impairment, and behavioral changes observed in AD patients (Querfurth and LaFerla, 2010). An increasing number of studies suggest that alterations in brain metabolic processes play an important role in AD pathogenesis, with brain insulin resistance recognized as an important feature of AD in both patients and in post-mortem tissue (Salkovic-Petrisic and Hoyer, 2007; Querfurth and LaFerla, 2010; Takeda et al., 2010; Bomfim et al., 2012; Talbot et al., 2012; Stanley et al., 2016).

Insulin and insulin-like growth factor (IGF) signaling in the brain regulate neuronal growth, repair, and synaptic maintenance (Stockhorst et al., 2004; van Dam and Aleman, 2004), and play an important role in learning and memory (Zhao et al., 2004). Reduced sensitivity to insulin and IGF signals in AD are observed across multiple levels of cell signal response, including reduced insulin receptor (IR) sensitivity, inhibition of secondary messengers (i.e., IR substrate-1), and decreased IR and IGF receptor (IGFR) expression (Watson and Craft, 2003; Rivera et al., 2005; Steen et al., 2005; Holscher and Li, 2010; de la Monte, 2012; Talbot et al., 2012; Freiherr et al., 2013; Stanley et al., 2016). Markers of insulin resistance are elevated in hippocampus, a region of the brain that has high levels of IRs and is affected in AD (Frölich et al., 1998; Talbot et al., 2012; Bedse et al., 2015). Furthermore, insulin sensitivity restoration, insulin, and IGF therapy have been suggested to improve cognitive performance and memory function in healthy humans, individuals with mild cognitive impairment (MCI), and AD patients (Reger et al., 2008; Bomfim et al., 2012; Bedse et al., 2015), protect neurons from amyloid-induced toxicity in primary cell culture studies (Mattson, 1997), and prevent tau hyperphosphorylation in animal models (Deng et al., 2009; Chen et al., 2013). Thus, in the context of insulin and IGF resistance, neurons may be more vulnerable to damage and death resulting from the pathological changes underlying AD.

IGF-I and IGF-II are growth factors secreted by tissues throughout the body including the brain, and are effectively stored outside of cells in complex with IGF binding proteins (IGFBPs) (Holly and Perks, 2006). Interaction with IGFBPs slows IGF clearance and regulates the activity of IGF on cells through a variety of mechanisms (Russo et al., 2005). Importantly, several studies have observed altered levels of IGFs and IGFBPs in the plasma and cerebrospinal fluid (CSF) of AD patients, further suggesting that the neuroprotective and synaptic maintenance effects elicited by IGF signaling may be altered in AD (Tham et al., 1993; Vardy et al., 2007; Salehi et al., 2008; Hertze et al., 2014; Åberg et al., 2015). While the relationship between plasma levels of IGFs and IGFBPs and pathological features of AD have been explored to some extent (Toledo et al., 2013; Lane et al., 2017; Mclimans et al., 2017), knowledge of how plasma and CSF IGFs as well as IGFBPs contribute to AD pathogenesis remains in its early stages with previous reports focusing on cross-sectional analyses of neuroimaging data or longitudinal analyses of specific candidate regions. Further, studies of CSF IGFBPs in AD conflict, with some reporting significant differences in levels of IGFBPs (Salehi et al., 2008; Hertze et al., 2014; Lane et al., 2017; Mclimans et al., 2017) while others report no difference (Åberg et al., 2015).

In this study, we investigated the relationship between CSF IGFBP-2 and multiple *in vivo* markers of AD pathology to expand upon recent findings suggesting that IGFBP-2 plays a role in AD progression and pathogenesis (Lane et al., 2017; Mclimans et al., 2017). To better understand how IGFBP-2 may impact AD pathogenesis, we further utilized gene expression data from transgenic mouse models of tauopathy and AD along with cell type specific expression from human and mouse to assess the relevance of IGFBP-2 dysregulation to neurodegeneration.

## Materials and Methods

### Participant Description

This study utilized samples from 300 individuals recruited for participation in the Alzheimer’s Disease Neuroimaging Initiative (ADNI) study with CSF measurements of IGFBP-2 as well as amyloid, tau, and p-tau available. At baseline, 89 were cognitively normal older adults (CN), 145 individuals were diagnosed with MCI, and 66 were clinically diagnosed with AD. Two-hundred and seventy-six of these individuals had at least two T1-weighted MR images available. The cohort is well-characterized and has been used in previously published studies (Desikan et al., 2013, 2014; Bonham et al., 2016). Clinical severity of symptoms in the MCI and AD groupings was measured using the Clinical Dementia Rating Scale Sum of Boxes (CDR-SB) Score (Morris, 1993) and Mini Mental State Exam (MMSE) (Folstein et al., 1975). A clinician diagnosed each participant using a structured protocol that utilized clinical judgment and neuropsychological tests. Briefly, controls were required to have normal memory function on the Logical Memory II subscale of the Wechsler Memory Scale – Revised (Wechsler, 1987), an MMSE score greater than 24, CDR total score equal to 0, and clinical determination that the individual was not significantly impaired in cognitive function or activities of daily living. Individuals with MCI were required to have abnormal memory function on the Logical Memory II subscale of the Wechsler Memory Scale – Revised, an MMSE greater than 24, CDR total score equal to 0.5, and clinical determination that the individual’s general cognition and functional performance was not impaired enough to make a diagnosis of AD. Finally, individuals with AD were required to have abnormal memory function on the Logical Memory II subscale of the Wechsler Memory Scale – Revised, an MMSE between 20 and 26, CDR total score equal to 0.5 or 1.0, and judgment by a clinician that the individual met NINCDS/ADRDA criteria for probable AD (McKhann et al., 1984). Informed and written consent was obtained from all study participants and the University of California, San Francisco institutional review board approved all aspects of this study.

### CSF Biomarker Measurements

The AlzBio3 Luminex xMAP immunoassay (Innogenetics, Ghent, Belgium) was used to measure CSF amyloid β_1__-42_ (amyloid), total tau (t-tau), p-tau_181p_ (p-tau) as described previously (Shaw et al., 2009; Kang et al., 2012). This method uses monoclonal antibodies specific for amyloid, t-tau, and p-tau. The monoclonal antibodies are chemically bonded to color-coded beads along with analyte-specific detector antibodies. Baseline CSF IGFBP-2 levels were measured using the Human DiscoveryMAP panel developed by Rules Based Medicine (Myriad RBM; Austin, TX, United States). The Human DiscoveryMAP panel is commercially available and measures a collection of metabolic, lipid, inflammatory, and other AD-relevant indicators. At the time this panel was used in the ADNI cohort, IGFBP-2 was the only IGF-related analyte in the panel. A full list of the measured metabolites is available through Myriad RBM. The CSF measurements in the immunoassay panel were processed and normalized according to previously described methods (Craig-Schapiro et al., 2011; Siuciak, 2011). Briefly, Myriad RBM used a Luminex 100 instrument for the measurements and analyzed the resulting data using proprietary software. The ADNI staff checked analyte distributions for normality using Box-Cox analyses and, if needed, log10 transformed the data to achieve an approximately normal distribution.

### Genotyping and Gene Expression Data

*APOE* status in the ADNI cohort was determined using DNA extracted by Cogenics (now Beckman Coulter Inc., Pasadena, CA, United States) from a 3 mL aliquot of EDTA blood.

We evaluated *IGFBP2* expression using AD and tau transgenic mouse model data from mouseac (Matarin et al., 2015)^1^. Briefly, microarray gene expression data was collected from three brain regions (cortex, hippocampus, and cerebellum) from wild-type, TASTPM (TAS10 × TPM AD mouse models; APPswe × PS1.M1466V), and P301L-tau transgenic mice. Brain tissue samples were at 2, 4, 8, and 18 months of age and raw expression levels were normalized using a log_2_ transformation; all samples were quantile normalized together.

To better understand the cell type-specific expression of *IGFBP2*, we utilized two publicly available RNA sequencing expression datasets examining several cell-types commonly found in the central nervous system (CNS). For additional details on sample processing and cohort characteristics, please see Zhang et al. (2014, 2016) and Bennett et al. (2016).

### Neuroimaging Data

One thousand one-hundred and sixteen T1-weighted MRI scans were processed using a quantitative volume and surface-based analysis technique which automatically segments scans into regions-of-interest (ROI) (Fischl et al., 2002; Desikan et al., 2006). The MRI scans were checked for quality and corrected for spatial distortion. All MRI scans were processed using Quarc (Quantitative Anatomical Regional Change), a modified version of the FreeSurfer pipeline designed to accurately estimate longitudinal changes in brain structure (Fennema-Notestine et al., 2007; Mcevoy et al., 2009; Holland and Dale, 2011; Holland et al., 2012). Cortical and subcortical ROIs were delineated using previously described automated parcellation and segmentation methods (Fischl et al., 2002; Desikan et al., 2006). The techniques used to estimate longitudinal sub-regional change for serial MRI scans are previously described (Holland and Dale, 2011). Briefly, Quarc utilizes non-linear registration of serial MR images to generate a deformation field that aligns both large and small structures with high fidelity. Volumetric changes are estimated as a percent change from the deformation field within a specified ROI. Quarc has been shown to be more a more sensitive measure of change over time compared to other measures of longitudinal brain atrophy such as the longitudinal FreeSurfer pipeline, TBM, and BSI (Holland et al., 2012). Quarc has been utilized extensively and has been shown to correlate closely with biomarkers of clinical progression (Desikan et al., 2011, 2013, 2014). We examined all 34 cortical regions of interest in the Desikan Killiany Atlas (Desikan et al., 2006) along with hippocampus and amygdala. For each region of interest, the change rate in the right and left structures was averaged.

### Statistical Analysis

#### Demographic Comparisons

Discrete and continuous demographic variables were compared across diagnostic groups using chi-squared and ANOVA analyses, respectively.

#### Cross-Sectional CSF t-tau and p-tau Analyses

Linear models were used to test for an association between IGFBP-2 and t-tau and IGFBP-2 with p-tau. We controlled for age, sex, education, CDR-SB score, and *APOE* 𝜀4 carrier status.

#### Neuroimaging Analyses

Linear mixed effects models were used to assess the relationship between IGFBP-2 levels and longitudinal gray matter atrophy controlling for baseline and time interactions of age, sex, education, baseline CDR-SB score, and *APOE* 𝜀4 carrier status.

We used the following linear mixed effect model:

$$\begin{array}{l} \Delta ROI\;Volume=\beta_{0}+\beta_{1}\Delta t+\beta_{2}IGFBP-2*\Delta t+\beta_{3}\text{Age}*\\ \Delta t+\beta_{4}\text{Sex*}\Delta t+\beta_{5}Education*\Delta t+\beta_{6}CDR-SB*\Delta t+\\ \beta_{7}APOE\varepsilon 4*\Delta t+e \end{array}$$

For neuroanatomical regions that showed volume change significantly predicted by IGFBP-2 levels only, we also assessed whether IGFBP-2 levels were associated with atrophy independent of baseline t-tau levels by adding the relevant terms to the original mixed effects model as follows:

$$\begin{array}{l} \Delta ROI\;Volume=\beta_{0}+\beta_{1}\Delta t+\beta_{2}IGFBP-2*\Delta t+\beta_{3}\text{Age}*\\ \Delta t+\beta_{4}\text{Sex*}\Delta t+\beta_{5},Education*\Delta t+\beta_{6}CDR-SB*\Delta t+\\ \beta_{7}APOE\varepsilon 4*\Delta t+\beta_{8}t-tau*\Delta t+e \end{array}$$

The results of these analyses were used in statistical mediation analyses. We used the coefficients to perform the Aroian test as described by Preacher and Hayes (2004).

#### Gene Expression Analyses

ANOVA was used to determine whether *IGFBP2* expression varied between wild-type and tau transgenic mice in hippocampus and cortex. We chose not to analyze cerebellar expression because this region is generally spared in AD and the tau transgenic mouse models we used do not display cerebellar pathology.

## Results

### Cohort Description

Data from 300 individuals identified as CN, MCI, or AD were included in this study (**Table 1**). The cohort was balanced with respect to age and education but differed by sex (*p* = 7.35 × 10^-3^). As expected, there were significant differences by diagnosis for *APOE* 𝜀4 distribution, CSF amyloid, CSF t-tau, and CSF p-tau. CSF IGFBP-2 levels did not differ by diagnosis. For the 276 individuals with neuroimaging data, the demographic and biomarker composition was similar to the full cohort. The average number of scans per participant across the entire cohort was about 4 (CN: 3.97 ± 0.9, MCI: 4.40 ± 1.1, AD: 3.4 ± 0.8) with an average follow-up time per participant of 2.1 years (CN: 2.39 ± 0.8, MCI: 2.16 ± 0.7, AD: 1.73 ± 0.6). A histogram depicting the timing of follow-up of scans relative to the baseline visit is provided in Supplementary Figure 1.

**Table 1:** Demographic information for participants included in the analysis.

	CN	MCI	AD	*p*-Value
*N*	89	145	66	NA
*APOE* 𝜀4 Carrier (%)	23.5%	48.3%	56.1%	<0.001
Sex (% female)	50.6%	33.1%	28.8%	<0.01
Age (years)	75.7 ± 5.5	75.0 ± 7.2	74.9 ± 7.7	NS
Education (years)	15.6 ± 3.0	16.0 ± 3.0	15.0 ± 3.0	NS
CDR-SB	0.03 ± 0.1	1.56 ± 0.9	4.3 ± 1.6	<0.001
Aβ42 (pg/mL)	208.0 ± 52.9	160.7 ± 48.6	141.6 ± 35.6	<0.001
t-tau (pg/mL)	68.5 ± 26.8	104.2 ± 52.3	119.8 ± 54.6	<0.001
p-tau (pg/mL)	24.6 ± 12.9	36.0 ± 15.6	41.4 ± 20.5	<0.05
IGFBP-2 (ng/mL)	101.6 ± 18.0	104.8 ± 19.2	103.1 ± 18.8	NS

*Summary statistics for participants. Demographic, genetic, and biomarker data is summarized by diagnostic category. APOE 𝜀4 carrier includes those with 1 or 2 APOE 𝜀4 alleles. CDR-SB, Clinical Dementia Rating Sum of Boxes. Two-tailed p-values were from ANOVA (continuous traits) or chi-square (categorical values) tests by sex, gene carrier status. CN, normal control; MCI, mild cognitive impairment; AD, Alzheimer’s disease.*

### CSF IGFBP-2 Is Associated With CSF t-tau and p-tau Levels

**FIGURE 1:**
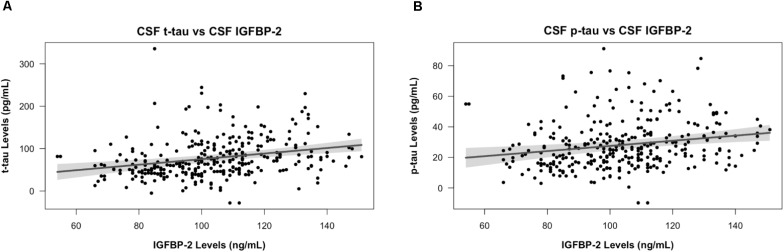
IGFBP-2 is associated with t-tau and p-tau in CSF. CSF t-tau **(A)** and p-tau **(B)** levels are plotted against CSF IGFBP-2 levels. IGFBP-2 levels are quality controlled and transformed as previously described (Siuciak, 2011). Higher levels of IGFBP-2 are associated with higher levels of t-tau and p-tau. The plotted points are partial residuals with 95% confidence bands provided in shading.

Across the entire cohort, IGFBP-2 was significantly associated with t-tau (β = 0.65 ± 0.15, *p* = 2.41×10^-5^) and p-tau (β = 0.17 ± 0.05, *p* = 1.61×10^-3^) in CSF, with higher levels of IGFBP-2 associated with higher levels of t-tau and p-tau (**Figure 1** and **Table 2**). Within subgroups, the association between CSF t-tau and IGFBP-2 was significant after correction for multiple testing in CN only (β = 0.58 ± 0.16, *p* = 2.44×10^-4^), with MCI (β = 0.54 ± 0.23, *p* = 0.02) and AD (β = 0.79 ± 0.40, *p* = 0.05) having *p*-values above *p* = 0.017 (Supplementary Table 1). For CSF p-tau, there were fewer observations available and IGFBP-2 was significant in MCI only (β = 0.18 ± 0.07, *p* = 7.56 × 10^-3^). However, the direction of the estimated effect in both CN (β = 0.08 ± 0.08, *p* = 0.32) and AD (β = 0.15 ± 0.15, *p* = 0.31) was consistent with the MCI grouping (Supplementary Table 1). There were no significant associations between IGFBP-2 levels and measures of CSF amyloid.

**Table 2:** Regression analysis results for predictors of cross sectional CSF p-tau and t-tau values in full cohort and by disease and meta-analysis groups.

Outcome	Variable	Estimate ± SE	*p*-Value
t-tau	Age	-0.63 ± 0.40	0.11
	Sex	18.33 ± 5.75	1.60 × 10^-3^
	CDR-SB	6.26 ± 1.50	3.89 × 10^-5^
	*APOE𝜀4* status	23.22 ± 5.40	2.31 × 10^-5^
	Education	0.28 ± 0.88	0.75
	IGFBP-2	0.65 ± 0.15	2.41 × 10^-5^
p-tau	Age	-0.28 ± 0.14	0.04
	Sex	2.40 ± 2.0	0.23
	CDR-SB	1.82 ± 0.52	5.30 × 10^-4^
	*APOE𝜀4* status	9.40 ± 1.87	8.59 × 10^-7^
	Education	0.06 ± 0.31	0.84
	IGFBP-2	0.17 ± 0.05	1.61 × 10^-3^

*IGFBP-2 is associated with t-tau and p-tau levels in CSF. Regression models used in cross-sectional CSF analyses of t-tau and p-tau are summarized. CSF IGFBP-2 was significantly associated with CSF t-tau and CSF p-tau. Higher levels of CSF IGFBP-2 were associated with higher levels of CSF t-tau and p-tau. The beta estimate (estimate) and accompanying standard error (SE) reflect the adjusted effect of each independent variable as a predictor of t-tau or p-tau. For all disease groups, the linear statistical model included as independent variables: age, sex, clinical disease rating sum of boxes (CDR-SB) APOE 𝜀4 carrier status, education, and IGFBP-2. All tests were two-tailed.*

### CSF IGFBP-2 Is Associated With Brain Atrophy in AD-Associated Regions

**FIGURE 2:**
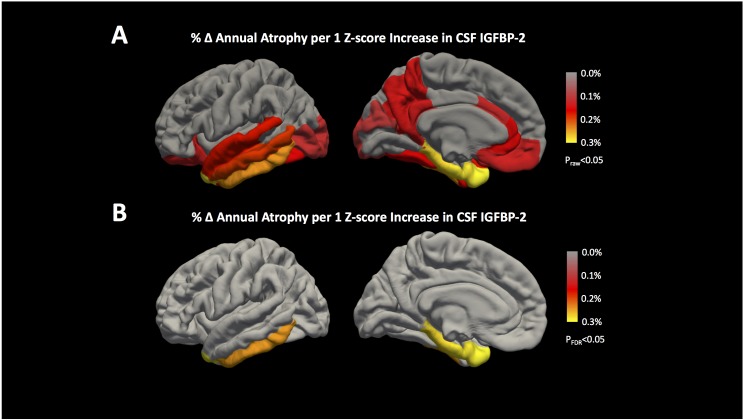
IGFBP-2 is associated with longitudinal atrophy in entorhinal, inferior temporal, temporal pole, and parahippocampal regions. Annualized changes in atrophy rate given a 1 *z*-score increase in CSF IGFBP-2 levels are shown for all 34 cortical regions included in the Desikan Killiany Atlas (Desikan et al., 2006). The results of our analyses are shown **(A)** before correction for multiple testing (*p*_raw_< 0.05) and **(B)** after correction for multiple testing (*p*_FDR_< 0.05) using the FDR method (Benjamini and Hochberg, 1995). Hippocampus and amygdala are not shown, but the results of these analyses are provided in Supplementary Table 2. IGFBP-2 levels are quality controlled and transformed as previously described (Siuciak, 2011). After correction for multiple testing, greater levels of CSF IGFBP-2 are associated with higher annual rates of atrophy in entorhinal, inferior temporal, temporal pole, and parahippocampal regions.

We next tested whether participants’ baseline CSF IGFBP-2 levels were associated with longitudinal volume change in all 34 Desikan Killiany cortical ROIs along with hippocampus and amygdala. At a raw *p* < 0.05, there were significant associations between CSF IGFBP-2 and atrophy in parahippocampal, entorhinal, inferior temporal, temporal pole, superior temporal, fusiform, isthmus cingulate, precuneus, rostral anterior cingulate, middle temporal, corpus callosum, caudal anterior cingulate, medial orbitofrontal, lateral occipital, and lateral orbitofrontal regions (**Figure 2A**). Additional details on the effect size and *p*-value for all regions are presented in Supplementary Table 2. After correction for multiple testing, CSF IGFBP-2 was significantly associated with atrophy in parahippocampal (β = -0.30 ± 0.06, *p* = 9.76 × 10^-5^), entorhinal (β = -0.31 ± 0.06, *p* = 1.15 × 10^-3^), inferior temporal (β = -0.26 ± 0.05, *p* = 3.85 × 10^-3^), and temporal pole (β = -0.32 ± 0.12, *p* = 3.83 × 10^-3^) regions (**Figure 2B**). All effects were consistent with greater CSF IGFBP-2 levels predicting greater atrophy over time.

Using mediation analysis, we found statistical evidence to suggest that CSF t-tau levels could partially explain the effects of IGFBP-2 on brain volume. We used the Aroian test to statistically assess whether the relationship between CSF IGFBP-2 and CSF t-tau mediated atrophy in parahippocampal, entorhinal, temporal pole, and inferior temporal regions. CSF t-tau statistically mediated the effect of CSF IGFBP-2 on atrophy in parahippocampal (*p* = 0.007), entorhinal (*p* = 0.01), inferior temporal (*p* = 0.003), and temporal pole (*p* = 0.02) regions.

### *Igfbp2/IGFBP2* Is Differentially Expressed in Transgenic Mice and Selectively Expressed in Astrocytes

**FIGURE 3:**
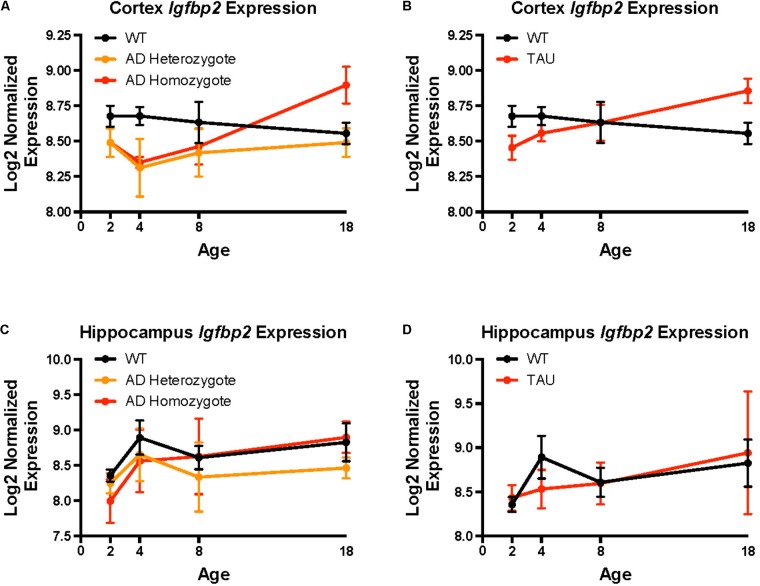
*Igfbp2* expression levels in mouse models of neurodegeneration. Mouse *Igfbp2* expression from Mouseac (www.mouseac.org) is plotted at ages 2, 4, 8, and 18 months. **(A)** Cortex expression in the TASTPM (AD) mouse model. **(B)** Cortex expression in the P301L (TAU) transgenic mouse. **(C)** Hippocampus expression in the TASTPM (AD) mouse model. **(D)** Hippocampus expression in the P301L (TAU) transgenic mouse. The plotted points are mean expression at each age ± standard errors. Expression data was normalized using a log_2_ transformation and all samples were quantile normalized together. Please see Matarin et al. (2015) for additional details on experimental models and data processing.

*Igfbp2* was differentially expressed in both TASTPM (AD) and P301L tau-transgenic compared to wild-type (C57BL/6) mouse neuropathological data. In cortex, homozygote TASTPM AD mice displayed lower *Igfbp2* expression during early life and higher expression during late life compared to wild-type mice (**Figure 3A**; *F* = 9.28, *p* = 0.004). By contrast, heterozygote TASTPM AD mice showed consistently lower expression of *Igfbp2* across all ages compared to wild-type mice (**Figure 3A**; *F* = 6.26, *p* = 0.016). Cortical expression of *Igfbp2* in the P301L tau mouse model showed an expression pattern similar to TASTPM AD homozygotes, with greater expression at older ages (**Figure 3B**; *F* = 5.03, *p* = 0.029). In hippocampus, *Igfbp2* was not significantly different from wild-type expression in either TASTPM heterozygotes (**Figure 3C**; *F* = 0.21, *p* = 0.21) or homozygotes (**Figure 3C**; *F* = 0.88, *p* = 0.35). Similarly, *Igfbp2* was not significantly different in P301L tau transgenic mice compared to wild-type mice, with expression increasing over time in both genotypes (**Figure 3D**; *F* = 0.19, *p* = 0.67).

**FIGURE 4:**
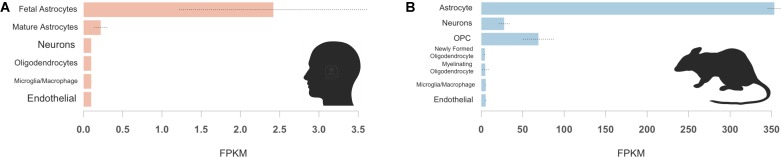
*IGFBP2* expression by cell type in human and mouse brain samples. Human *IGFBP2* **(A)** and mouse *Igfbp2* **(B)** expression is shown for selected CNS cell types available from (http://web.stanford.edu/group/barres_lab/brainseq2/brainseq2.html). In both humans and mice, astrocytes express *IGFBP2* most robustly oligodendrocyte precursor cell (OPC).

Finally, we assessed cell specificity of *IGFBP2* expression in the CNS. In both humans and mice, astrocytes expressed *IGFBP2/Igfbp2* most robustly (**Figure 4**). In human samples, fetal astrocytes expressed *IGFBP2* more highly than mature astrocytes (**Figure 4A**). In mice, oligodendrocyte progenitor cells and neurons were the next highest expressers of *Igfbp2* following astrocytes (**Figure 4B**).

## Discussion

Our study highlights several findings supporting the role of IGFBP-2 in tau-related AD pathobiology. Previous studies on AD-associated changes in CSF IGFBP-2 are conflicting; some studies demonstrate elevated levels of CSF IGFBP-2 in AD relative to age-matched controls (Salehi et al., 2008; Hertze et al., 2014), while others report similar findings to our study with no significant difference in CSF IGFBP-2 levels by diagnosis (Åberg et al., 2015; Lane et al., 2017). Furthermore, our finding that IGFBP-2 levels correlate with t-tau and p-tau in the CSF across the AD spectrum at baseline (**Table 2**) are in agreement with prior studies (Hertze et al., 2014; Åberg et al., 2015). Taken together with previous studies, our findings suggest that CSF IGFBP-2 levels associate with pathological burden across the spectrum of AD. Further experimental studies are required to elucidate a mechanistic connection between IGFBP-2 and tau pathology in AD.

A previous study reported an association between plasma IGFBP-2 levels and cross-sectional atrophy measured by SPARE-AD score in a subset of healthy controls, MCI, and AD participants from ADNI, but found no association between plasma IGFBP-2 levels and atrophy in specific brain regions (Toledo et al., 2013). More recent studies found that plasma IGFBP-2 was associated with hippocampal volumes as well as other volumetric and functional measures cross-sectionally, but not longitudinally (Lane et al., 2017; Mclimans et al., 2017). A recent report demonstrated a significant relationship (*p* = 0.023) between CSF amyloid and CSF IGFBP-2, potentially conflicting with our finding (Mclimans et al., 2017). The discrepancy between these findings and ours could be explained by differences in our covariate selection. For instance, we covaried for clinical severity (CDR-SB) score rather than for baseline diagnosis and included *APOE* 𝜀4 carrier status rather than *APOE* 𝜀4 dosage in all analyses. We found an association between baseline CSF IGFBP-2 levels and longitudinal changes in multiple non-hippocampal brain structures (Supplementary Table 2). Surprisingly, *Igfbp2* expression in transgenic mice was significantly different from wild type mice only in cortex and not in hippocampus, which may be why we (and other groups) failed to find a robust association between IGFBP-2 and longitudinal hippocampal atrophy. Additionally, it is possible that CSF IGFBP-2 represents a more proximal measure of IGF dysregulation in the brain relative to plasma levels, which may be more variable in a limited clinical cohort.

Insulin and IGF resistance due to type 2 diabetes in human patients significantly increases the risk of developing dementia, and is associated with regional brain atrophy (Leibson et al., 1997; Last et al., 2007; Stanley et al., 2016). Furthermore, impaired brain insulin and IGF signaling induced in rats by intracerebral injection of streptozotocin results in brain atrophy and neurodegeneration (Lester-Coll et al., 2006). While the exact role of IGFBP-2 in regulating IGF signaling in the brain is unclear, evidence in mice suggests that IGFBP-2 may inhibit IGF activity (Hoeflich et al., 1999). We provide statistical evidence that the association between CSF IGFBP-2 and entorhinal, parahippocampal, inferior temporal, and temporal pole atrophy may be related to intracerebral tau (estimated using CSF tau levels).

Tau dysregulation is a hallmark of AD pathology and contributes to neuronal cell loss (Querfurth and LaFerla, 2010). As impaired IGF signaling contributes to tau dysregulation (Bedse et al., 2015), the effect of IGFBP-2 on IGF signaling may explain how IGFBP-2 contributes to tau-related brain atrophy. Similarly, previous studies in primary neurons demonstrated that IGF-I prevents amyloid-induced increases in tau phosphorylation and cell death, and IGFBP-3 was able to inhibit these effects (Watanabe et al., 2015). Although IGFBP-2 may regulate IGF signaling in neurons differently than IGFBP-3, one might speculate that IGFBP-2 binds to IGFs, blocking IGF-mediated suppression of tau phosphorylation, leading to increased levels of p-tau and promoting neuronal damage and death.

A strength of our study is the use of a thoroughly characterized cohort of healthy aging control, MCI, and AD patients, a subset of which underwent multiple MRI scans and had baseline CSF protein levels quantified. Our findings utilized multiple data types and support a role for IGFBP-2 in AD pathobiology. However, our study is limited by its observational nature, which prevents us from establishing causative relationships. Additionally, the data from murine models of neurodegenerative disease only allowed for examination of whole cerebral cortex. However, our analyses using human data highlighted parahippocampal, entorhinal, inferior temporal, and temporal pole cortex as the regions whose atrophy is most associated with CSF IGFBP-2. Thus, we cannot easily compare the neuroanatomical relationships seen in our human data with the cross-sectional mouse data. As a correlative study, our findings suggest that CSF IGFBP-2 levels are related to AD, but do not carry any mechanistic implications. For example, although we have proposed an inhibitory role for IGFBP-2 on IGF signaling in the brain based on previous studies, others have indicated that IGFBP-2 may facilitate IGF signaling in the brain (Russo et al., 2005), and therefore elevated levels of IGFBP-2 in the brain may protect against AD pathogenesis. Thus, our results require follow-up in larger independent cohorts and experimental models to establish whether IGFBP-2 influences progression from normal cognition to AD and its potential biological role in AD pathogenesis.

In summary, we found that baseline IGFBP-2 levels correlate with t-tau and p-tau levels in the CSF of healthy aging control, MCI and AD patients. IGFBP-2 is associated with longitudinal rates of atrophy in AD-associated human cortical regions and its expression is dysregulated in transgenic mice with AD-relevant pathology. In both humans and mice, *IGFBP2/Igfbp2* is most highly expressed in astrocytes. Given the increasingly appreciated role of astrocytes in synaptic pruning during neurodegeneration (Liddelow et al., 2017), further studies may help to elucidate why this effect appears to be limited to non-hippocampal regions and how astrocyte-related metabolic disarray leads to tau pathology in AD.

## Author Contributions

LB conceived the study, conducted analyses, and drafted the manuscript. EG conceived the study, conducted analyses, and drafted the manuscript. NS drafted the manuscript and interpreted the data. DH processed MRI scans and provided guidance on image analysis. BM assisted with data interpretation. AD processed MRI scans, provided guidance on image analysis, and interpreted data. RD processed MRI scans, provided guidance on image as well as biomarker analyses, and drafted the manuscript. JY conceived the study, conducted analyses, and drafted the manuscript.

## Conflict of Interest Statement

RD is an editor for this special issue of Frontiers in Neuroscience. The remaining authors declare that the research was conducted in the absence of any commercial or financial relationships that could be construed as a potential conflict of interest.

## Footnotes

1 www.mouseac.org
