# Supplementary material for: Insulin-Like Growth Factor Binding Protein 2 Is Associated With Biomarkers of Alzheimer’s Disease Pathology and Shows Differential Expression in Transgenic Mice
Source: Front Neurosci. 2018 Jul 16;12:476. doi: 10.3389/fnins.2018.00476 (PMC6055061; doi:10.3389/fnins.2018.00476)
Supplement: Supplementary file 1 [file Table_1.DOCX]

**Supplementary Table 1.** Regression analysis results for predictors of cross sectional CSF p-tau and t-tau values in full cohort and by disease and meta-analysis groups

| **Outcome** | **Cohort** | **Variable** | **Estimate ± SE** | ***P*-Value** |
| --- | --- | --- | --- | --- |
| t-tau | All | Age | -0.63 ± 0.40 | 0.11 |
|  |  | Sex | 18.33 ± 5.75 | 1.60 x 10^-3^ |
|  |  | CDR-SB | 6.26 ± 1.50 | 3.89 x 10^-5^ |
|  |  | *APOE ε4* Status | 23.22 ± 5.40 | 2.31 x 10^-5^ |
|  |  | Education | 0.28 ± 0.88 | 0.75 |
|  |  | IGFBP-2 | 0.65 ± 0.15 | 2.41 x 10^-5^ |
|  | CN | Age | 0.61 ± 0.49 | 0.22 |
|  |  | Sex | 13.15 ± 5.72 | 0.02 |
|  |  | CDR-SB | -24.70 ± 22.94 | 0.28 |
|  |  | *APOE ε4* Status | 14.76 ± 6.21 | 0.02 |
|  |  | Education | 0.67 ± 0.94 | 0.48 |
|  |  | IGFBP-2 | 0.58 ± 0.16 | 4.42 x 10^-4^ |
|  | MCI | Age | -0.23 ± 0.60 | 0.70 |
|  |  | Sex | 19.06 ± 9.52 | 0.05 |
|  |  | CDR-SB | 3.76 ± 4.56 | 0.41 |
|  |  | *APOE ε4* Status | 29.29 ± 8.01 | 3.56 x 10^-4^ |
|  |  | Education | -0.65 ± 1.31 | 0.62 |
|  |  | IGFBP-2 | 0.54 ± 0.23 | 0.02 |
|  | AD | Age | -1.57 ± 0.89 | 0.08 |
|  |  | Sex | 32.31 ± 14.58 | 0.03 |
|  |  | CDR-SB | -1.23 ± 4.19 | 0.77 |
|  |  | *APOE ε4* Status | -3.86 ± 14.95 | 0.80 |
|  |  | Education | 0.55 ± 2.39 | 0.82 |
|  |  | IGFBP-2 | 0.79 ± 0.40 | 0.05 |
| **Outcome** | **Cohort** | **Variable** | **Estimate ± SE** | ***P*-Value** |
| p-tau | All | Age | -0.28 ± 0.14 | 0.04 |
|  |  | Sex | 2.40 ± 2.0 | 0.23 |
|  |  | CDR-SB | 1.82 ± 0.52 | 5.30 x 10^-4^ |
|  |  | *APOE ε4* Status | 9.40 ± 1.87 | 8.59 x 10^-7^ |
|  |  | Education | 0.06 ± 0.31 | 0.84 |
|  |  | IGFBP-2 | 0.17 ± 0.05 | 1.61 x 10^-3^ |
|  | CN | Age | 0.51 ± 0.25 | 0.04 |
|  |  | Sex | 1.06 ± 2.84 | 0.71 |
|  |  | CDR-SB | -11.14 ± 11.41 | 0.33 |
|  |  | *APOE ε4* Status | 9.65 ± 3.09 | 2.46 x 10^-3^ |
|  |  | Education | 0.31 ± 0.47 | 0.51 |
|  |  | IGFBP-2 | 0.08 ± 0.08 | 0.32 |
|  | MCI | Age | -0.10 ± 0.17 | 0.57 |
|  |  | Sex | 4.29 ± 2.77 | 0.12 |
|  |  | CDR-SB | 1.38 ± 1.33 | 0.3 |
|  |  | *APOE ε4* Status | 10.74 ± 2.33 | 8.57 x 10^-6^ |
|  |  | Education | -0.25 ± 0.38 | 0.52 |
|  |  | IGFBP-2 | 0.18 ± 0.07 | 7.56 x 10^-3^ |
|  | AD | Age | -0.94 ± 0.34 | 7.24 x 10^-3^ |
|  |  | Sex | 3.27 ± 5.57 | 0.56 |
|  |  | CDR-SB | -1.0 ± 1.60 | 0.53 |
|  |  | *APOE ε4* Status | -1.24 ± 5.71 | 0.83 |
|  |  | Education | -0.03 ± 0.91 | 0.97 |
|  |  | IGFBP-2 | 0.15 ± 0.15 | 0.31 |

**Supplementary Table 1 Legend. *IGFBP-2 is associated with t-tau and p-tau levels in CSF*.** Regression models used in cross-sectional CSF analyses of t-tau and p-tau are summarized. CSF IGFBP-2 was significantly associated with CSF t-tau and CSF p-tau in cases and controls. Higher levels of CSF IGFBP-2 were associated with higher levels of CSF t-tau and p-tau. For CSF t-tau, the relationship was replicated within each diagnostic grouping. For CSF p-tau, there were fewer observations available and CSF IGFBP-2 was significant in MCI grouping only. The beta estimate (estimate) and accompanying standard error (SE) reflect the adjusted effect of each independent variable as a predictor of t-tau or p-tau. For all disease groups, the linear statistical model included as independent variables: age, sex, clinical disease rating sum of boxes (CDR-SB) *APOE ε*4 carrier status, education, and IGFBP-2. All tests were two-tailed.
