# Supplementary material for: Insulin-Like Growth Factor Binding Protein 2 Is Associated With Biomarkers of Alzheimer’s Disease Pathology and Shows Differential Expression in Transgenic Mice
Source: Front Neurosci. 2018 Jul 16;12:476. doi: 10.3389/fnins.2018.00476 (PMC6055061; doi:10.3389/fnins.2018.00476)
Supplement: Supplementary file 2 [file Table_2.docx]

**Supplementary Table 2.** Longitudinal changes in brain volumes are associated with baseline CSF IGFBP-2

| FreeSurfer ROI | β | SE | P | P_FDR_ |
| --- | --- | --- | --- | --- |
| Parahippocampal | -0.30 | 0.06 | 9.76E-05 | 3.51E-03 |
| Entorhinal | -0.31 | 0.06 | 1.15E-03 | 0.02 |
| Inferior temporal | -0.26 | 0.05 | 3.85E-03 | 0.03 |
| Temporal pole | -0.32 | 0.12 | 3.83E-03 | 0.03 |
| Superior temporal | -0.18 | 0.07 | 7.55E-03 | 0.05 |
| Fusiform | -0.16 | 0.07 | 0.01 | 0.06 |
| Isthmus cingulate | -0.14 | 0.07 | 0.01 | 0.06 |
| Precuneus | -0.15 | 0.06 | 0.01 | 0.06 |
| Rostral anterior cingulate | -0.16 | 0.06 | 0.01 | 0.06 |
| Middle temporal | -0.20 | 0.04 | 0.02 | 0.07 |
| Corpus callosum | -0.12 | 0.09 | 0.02 | 0.08 |
| Caudal anterior cingulate | -0.13 | 0.09 | 0.03 | 0.08 |
| Medial orbitofrontal | -0.13 | 0.07 | 0.03 | 0.08 |
| Lateral occipital | -0.12 | 0.07 | 0.04 | 0.10 |
| Lateral orbitofrontal | -0.14 | 0.06 | 0.04 | 0.10 |
| Cuneus | -0.10 | 0.08 | 0.05 | 0.10 |
| Inferior parietal | -0.14 | 0.05 | 0.05 | 0.11 |
| Posterior cingulate | -0.11 | 0.06 | 0.06 | 0.11 |
| Superior frontal | -0.12 | 0.07 | 0.06 | 0.11 |
| Hippocampus | -0.19 | 0.10 | 0.06 | 0.11 |
| Superior parietal | -0.13 | 0.06 | 0.08 | 0.13 |
| Lingual | -0.07 | 0.06 | 0.09 | 0.15 |
| Rostral middle frontal | -0.11 | 0.06 | 0.11 | 0.17 |
| Parstriangularis | -0.09 | 0.07 | 0.15 | 0.21 |
| Pericalcarine | -0.06 | 0.06 | 0.15 | 0.21 |
| Supramarginal | -0.11 | 0.07 | 0.15 | 0.21 |
| Pars opercularis | -0.09 | 0.06 | 0.17 | 0.22 |
| Transverse temporal | -0.07 | 0.06 | 0.26 | 0.34 |
| Paracentral | -0.06 | 0.09 | 0.34 | 0.41 |
| Pars orbitalis | -0.07 | 0.06 | 0.33 | 0.41 |
| Postcentral | -0.05 | 0.04 | 0.38 | 0.44 |
| Bankssts | -0.05 | 0.11 | 0.48 | 0.54 |
| Frontal pole | -0.08 | 0.08 | 0.50 | 0.55 |
| Amygdala | -0.05 | 0.10 | 0.60 | 0.64 |
| Caudal Middle Frontal | -0.03 | 0.08 | 0.73 | 0.75 |
| Precentral | -0.01 | 0.06 | 0.89 | 0.89 |

**Supplementary Table 2 Legend. *CSF IGFBP-2 is associated with entorhinal, temporal pole, inferior temporal, and parahippocampal atrophy.*** Linear mixed effects regression models used to assess the relationship between IGFBP-2 and brain volumes are summarized. For each region of interest, both the right and left structure were used in the linear mixed effects model to assess the relationship between CSF IGFBP-2 levels and longitudinal grey matter atrophy controlling for baseline age, sex, education, baseline CDR-SB score, and *APOE* ε4 carrier status. Longitudinal sub-regional changes for 1,116 T1-weighted, serial MRI scans were estimated using methods that correlate closely with biomarkers of clinical progression. Scans were processed using quantitative volume and surface-based analysis techniques, which automatically segment scans into regions-of-interest (ROI). After correction for multiple testing, there were significant associations in the entorhinal, temporal pole, inferior temporal, and parahippocampal regions. The β estimate and accompanying standard error (SE) reflect the adjusted effect of each independent variable as a predictor of volumetric change in the region of interest. All tests were two-tailed.
