# Supplementary material for: Insulin-Like Growth Factor Binding Protein 2 Is Associated With Biomarkers of Alzheimer’s Disease Pathology and Shows Differential Expression in Transgenic Mice
Source: Front Neurosci. 2018 Jul 16;12:476. doi: 10.3389/fnins.2018.00476 (PMC6055061; doi:10.3389/fnins.2018.00476)
Supplement: Supplementary file 3 [file Image_1.pdf]

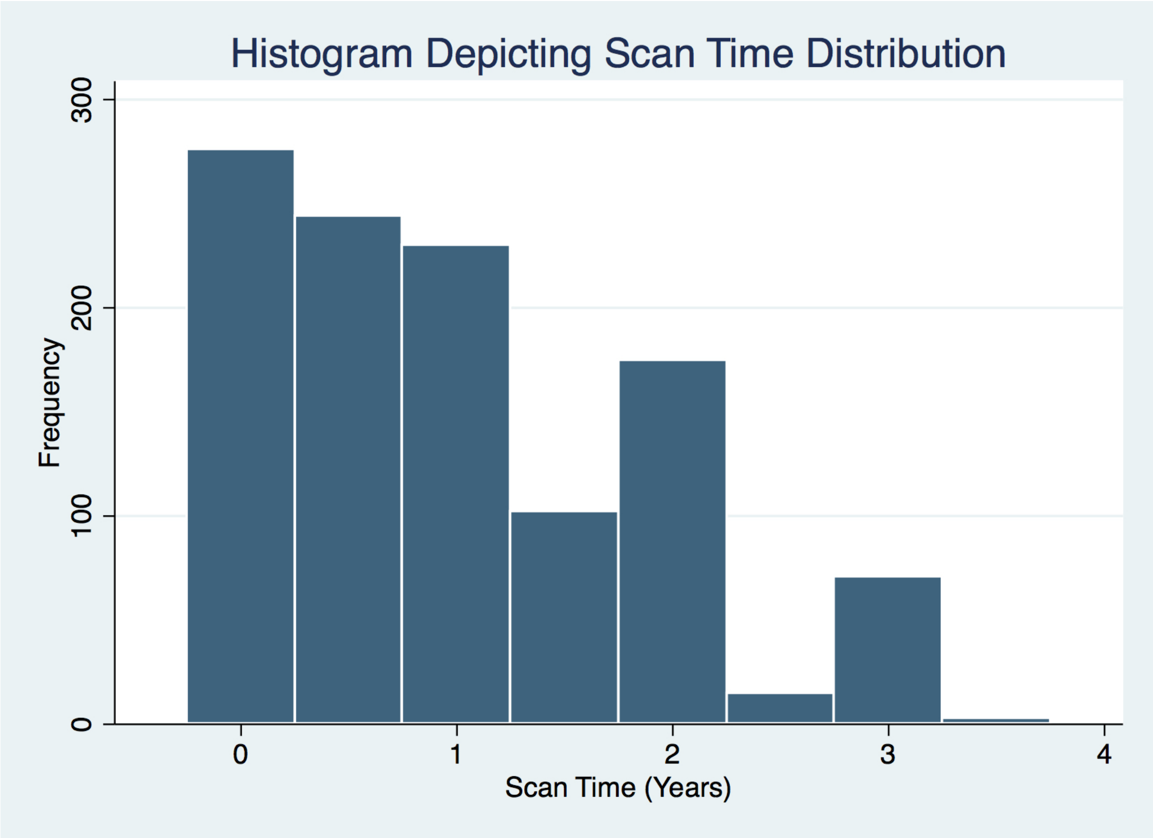

Supplementary Figure 1. Histogram of MRI scans across all participants with neuroimaging data in this study.
